# Disulfide stabilization of human norovirus GI.1 virus-like particles focuses immune response toward blockade epitopes

**DOI:** 10.1038/s41541-020-00260-w

**Published:** 2020-12-14

**Authors:** Raffaello Verardi, Lisa C. Lindesmith, Yaroslav Tsybovsky, Jason Gorman, Gwo-Yu Chuang, Caitlin E. Edwards, Paul D. Brewer-Jensen, Michael L. Mallory, Li Ou, Arne Schön, Wei Shi, Ena S. Tully, George Georgiou, Ralph S. Baric, Peter D. Kwong

**Affiliations:** 1 Vaccine Research Center, National Institute of Allergy and Infectious Diseases, National Institutes of Health, Bethesda, 20892 MD USA; 2 Department of Epidemiology, University of North Carolina, Chapel Hill, 27599 NC USA; 3 Electron Microscopy Laboratory, Cancer Research Technology Program, Leidos Biomedical Research, Inc., Frederick National Laboratory for Cancer Research, Frederick, 21702 MD USA; 4 Department of Biology, Johns Hopkins University, Baltimore, 21218 MD USA; 5 Department of Chemical Engineering, University of Texas at Austin, Austin, 78712 TX USA

**Keywords:** Protein vaccines, Viral infection

## Abstract

Human noroviruses are non-enveloped, single-strand RNA viruses that cause pandemic outbreaks of acute gastroenteritis. A bivalent vaccine containing GI.1 and GII.4 virus-like particles (VLPs) has been shown to be safe and highly immunogenic, but its efficacy and durability have been limited. Here, we show that norovirus GI.1 VLPs are unstable and contain a substantial fraction of dissociated VLP components. Broadly reactive, non-neutralizing antibodies isolated from vaccinated donors bound to the dissociated components, but not to the intact VLPs. Engineering of interprotomer disulfide bonds within the shell domain prevented disassembly of the VLPs, while preserving antibody accessibility to blockade epitopes. Without adjuvant, mice immunized with stabilized GI.1 VLPs developed faster blockade antibody titers compared to immunization with wild-type GI.1 VLPs. In addition, immunization with stabilized particles focused immune responses toward surface-exposed epitopes and away from occluded epitopes. Overall, disulfide-stabilized norovirus GI.1 VLPs elicited improved responses over the non-disulfide-stabilized version, suggesting their promise as candidate vaccines.

## Introduction

Noroviruses are single-stranded RNA viruses that cause pandemic outbreaks of acute gastroenteritis^1^. They are the primary viral agents of food borne diseases worldwide, and they are responsible for >200,000 deaths per year (mostly among infants and elderly in developing countries)^2^. Due to high infectivity, noroviruses are also a significant threat to transplant patients and immunocompromised individuals^3,4^. Although discovered over 50 years ago, no vaccine nor drugs (antibodies or small molecules) are currently licensed to prevent or treat norovirus infections^5^.

In the absence of a widely available tissue culture system that can sustain replication of human noroviruses, virus-like particles (VLPs) have been used as a surrogate to study the capsid’s structural features and as immunogens to elicit protective humoral responses^6–8^. Recently, VLPs have emerged as valuable immunogens for the elicitation of durable protective serological memory^9,10^. The most advanced norovirus vaccine candidate is a bivalent formulation comprising a mixture of GI.1 and GII.4 VLPs, administered intramuscularly^11–13^. Results from phase IIb clinical trials have revealed that the vaccine is highly immunogenic and can elicit high titers of blockade antibodies^11,14^. However, the vaccine is <50% protective against GI.1 challenge and, at least for GII.4, serum blockade titers wane rapidly following immunization^15,16^. In a recent study, ten antibodies were isolated from three donors after immunization with the bivalent vaccine^17^. The antibodies could be classified into two broad classes: (1) cross-reactive (capable of binding VLPs from genogroups I and II) but non-neutralizing, and (2) genotype-specific (only targeting GII.4 variants) and neutralizing. Structural analysis of the antibody Fab fragments in complex with the P domain of GII.4 (2002 Farmington Hills strain) revealed cross GI and GII reactive antibodies to target a site on the P domain that would be completely buried in the context of the intact viral particle^17^. Similar antibodies (herein referred to as occluded-site antibodies) have been previously observed after immunization, with norovirus VLPs in mice^18–20^. Two questions arise: how can such antibodies be elicited, and can vaccine performance be improved by preventing their elicitation? To shed light on these issues, we investigated the interaction between GI.1 norovirus VLPs and one antibody belonging to each class. First, we observed that GI.1 VLP preparations contained dissociated VP1 components even after extensive purification, with a substantial amount of VP1 dimers. The cross-reactive, but non-neutralizing, antibody A1227 interacted strongly with VP1 dimers, but not with intact particles; in contrast, the GI.1 specific and blockade antibody 512 could bind to VP1 dimers, as well as to intact particles. Structure-based design of interprotomer disulfide bonds resulted in GI.1 VLPs that did not dissociate and did not bind occluded-site antibodies. Crucially, stabilization did not compromise accessibility to known neutralizing epitopes. Finally, immunization with stabilized VLPs elicited blockade titers more rapidly and appeared to focus the immune responses toward accessible (and potentially neutralizing) epitopes. Together, our data suggest interprotomer disulfide stabilization as a possible avenue to improve VLP-based norovirus vaccines.

## Results

### GI.1 norovirus VLP preparations contain VP1 dimers and other oligomers that expose occluded-site epitopes

**Fig. 1:**
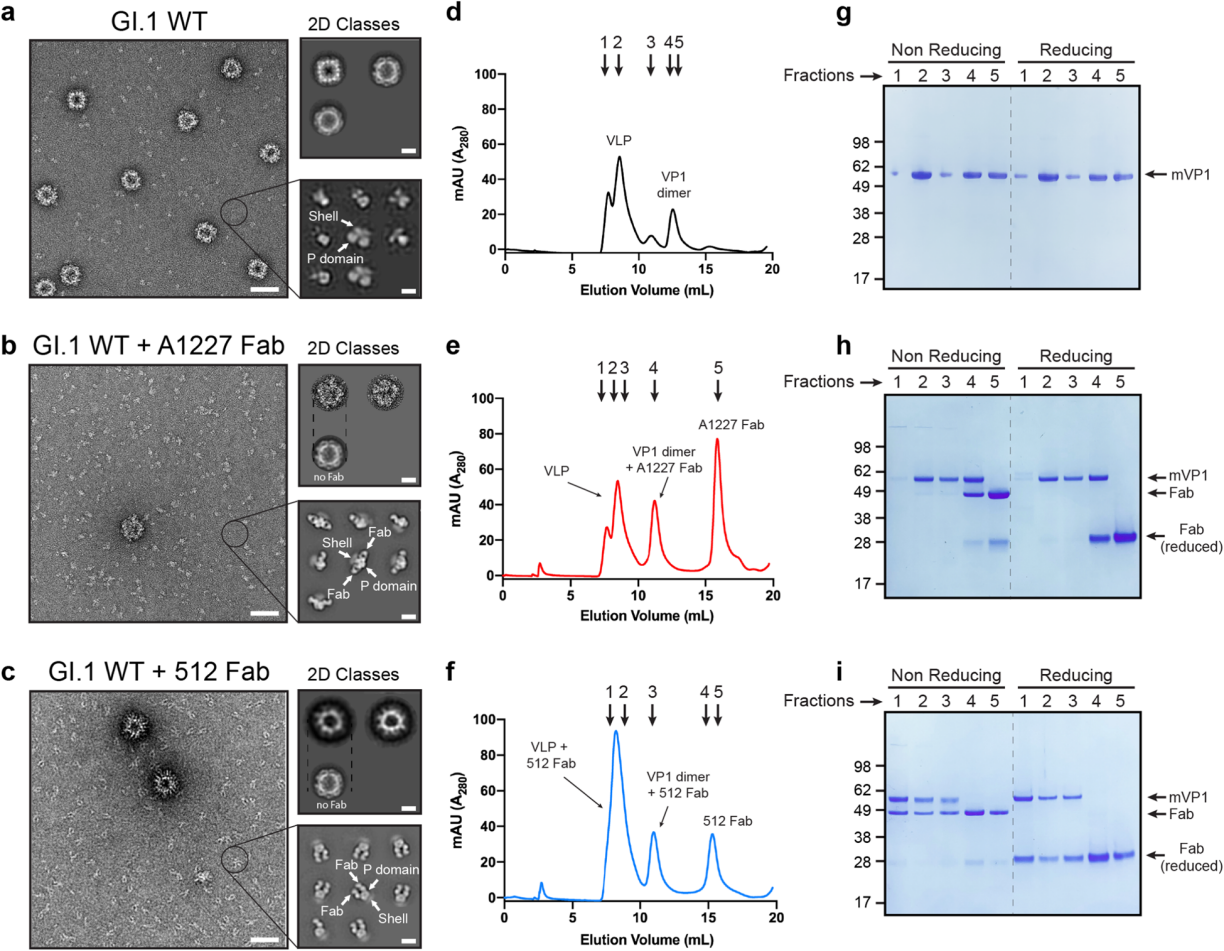
Dissociation of GI.1 norovirus VLPs leads to exposure of occluded epitopes. **a** Representative micrograph of negatively stained GI.1 WT VLPs. 2D class averages of small objects are shown in the bottom right corner, and 2D class averages of intact VLPs in the top right corner. Notice the presence of dissociated VP1 dimers and larger oligomers. The shell and P domain are indicated by white arrows. **b** Representative micrograph of GI.1 WT after incubation with A1227 Fab fragment. 2D class averages of small objects show 2 Fabs bound to each VP1 dimer. Intact particles do not show any A1227 Fab bound. **c** Representative micrograph of GI.1 WT after incubation with 512 Fab fragment. Class averages show VP1 dimers bound to two Fabs and intact VLPs decorated with a layer of 512 Fabs. **d**–**f** Analytical size-exclusion chromatography of **d** GI.1 WT, **e** GI.1 WT + A1227 Fab, and **f** GI.1 WT + 512 Fab. In all cases, the peak at 8.5 mL elution volume corresponds to void volume. **g** SDS–PAGE analysis of fractions from chromatogram in **d**. **h** SDS–PAGE analysis of fractions from chromatogram in **e**. Fraction 4 contains equal amounts of VP1 and A1227 Fab, indicative of 1:1 complex (1 Fab: 1 VP1), while fraction 5 contains free A1227 Fab. **i** SDS–PAGE analysis of chromatogram in **f**. 512 Fab is present in void peak (decorated VLPs) and in 1:1 complex with VP1 dimers (fraction 3). Free 512 Fab is found in fractions 4 and 5. In all gels, the non-covalent VP1 dimer runs as a monomer (mVP1). Scale bars are 100, 20, and 10 nm for micrographs, 2D classes of VLPs, and 2D classes of small proteins, respectively.

Serological analysis of antibody repertoires from humans vaccinated with a cocktail of GI.1 and GII.4 norovirus VLPs has identified broadly reactive but non-neutralizing antibodies^17^. Binding data showed that one antibody belonging to this class (A1227) could bind to several VLPs from genogroup I and II, but no neutralization was observed against infectious GII.4 noroviruses in an organoid system^17^. The crystal structure of the A1227 Fab fragment bound to the GII.4 P domain revealed that the epitope for this antibody would be inaccessible, when mapped on the surface of an intact GI.1 norovirus VLP (the only high-resolution structure of a human norovirus VLP available at the time)^17^. To determine how A1227 could interact with norovirus VLPs, we expressed the VP1 protein from GI.1 Norwalk strain (herein referred to as GI.1 WT) in insect cells and purified the self-assembled particles using a combination of gradient ultra-centrifugation and size-exclusion chromatography (Supplementary Fig. 1). We first analyzed the GI.1 WT VLPs using negative staining electron microscopy (NS-EM). Although most of the particles were correctly formed and showed the expected diameter, analysis of 2D class averages of smaller proteins in the background revealed the presence of VLP components that appeared to be mostly VP1 dimers (Fig. 1a).

Next, we incubated A1227 Fab fragment with purified GI.1 WT VLPs. Surprisingly, after adding A1227 Fab (at a molar ratio of 1:2—VP1:Fab), we only observed complexes containing one VP1 dimer and two Fabs (Fig. 1b). Although not enough intact VLPs were available to produce 2D class averages, it appeared that A1227 Fab did not bind to intact particles (Fig. 1b). In contrast, after addition of the GI.1 neutralizing antibody 512 (Fab fragment), which has been shown to bind to the P domain near the receptor-binding site at the apex of the P domain^21^, we saw VLPs decorated with 512 Fabs, as well as VP1 dimers bound to two Fabs (Fig. 1c). Fitting of the A1227/GII.4c P domain crystal structure on the low-resolution 3D reconstruction of the complexes from NS-EM data showed that the Fabs bind to the GI.1 VP1 dimers with a similar angle as to the GII.4 P domain dimer (Supplementary Fig. 2a), confirming that the epitope on the GI.1 P domain is similar for both GI.1 and GII.4 genotypes. The same results were obtained by fitting the crystal structure of GI.1 P domain/512 Fab complex onto the 3D map of the corresponding VP1/Fab complex (Supplementary Fig. 2b).

To rule out the possibility that VLP heterogeneity was due to artifacts from grid preparation for NS-EM, we used analytical size-exclusion chromatography to assess particle dissociation and interaction with antibodies. In the absence of antibody A1227, GI.1 VLPs eluted in two main peaks (Fig. 1d), the first (fractions 1 and 2) corresponding to the void volume, indicative of non-dissociated VLPs and the second ~13 mL elution volume (fractions 4 and 5), consistent with VP1 dimers. Integration of the chromatogram indicates that ~20% of the sample is present as VP1 dimers. SDS–PAGE analysis confirmed the presence of VP1 protein in both peaks (Fig. 1g). When VLPs were incubated with A1227 Fab, the size and position of the void peak (fractions 1–3) did not change, while the VP1 dimer peak shifted by 2 mL (fraction 4), indicative of complex formation, while the peak at 16 mL was consistent with free Fab (Fig. 1e). Analysis of the fractions by SDS–PAGE revealed that A1227 Fab is found in the VP1 dimer peak (fraction 4) and as free Fab (fraction 5), but not in the fractions with intact particles (Fig. 1h). These results were consistent with the NS-EM data showing no interaction between GI.1 VLPs and A1227, but strong interaction between A1227 and dissociated VP1 dimers. Finally, the addition of 512 Fab to GI.1 VLPs also resulted in three peaks (Fig. 1f, i). Antibody 512 Fabs were found in complex with the dissociated VP1 dimers (fraction 3) and in free form (fraction 4 and 5), but also in the void peak (fractions 1 and 2), indicative of Fab/VLP interaction.

In summary, expression of norovirus GI.1 VP1 protein in insect cells led to the production of intact VLPs and a small population of dissociated particle components (primarily VP1 dimers). Cross-reactive but non-neutralizing antibody A1227 bound only the dissociated VP1 dimers, while GI.1-specific and blockade antibody 512 bound to intact particles, as well as to VP1 dimers.

### Structure-based design of disulfide bonds between VP1 monomers leads to stabilized particles

**Fig. 2:**
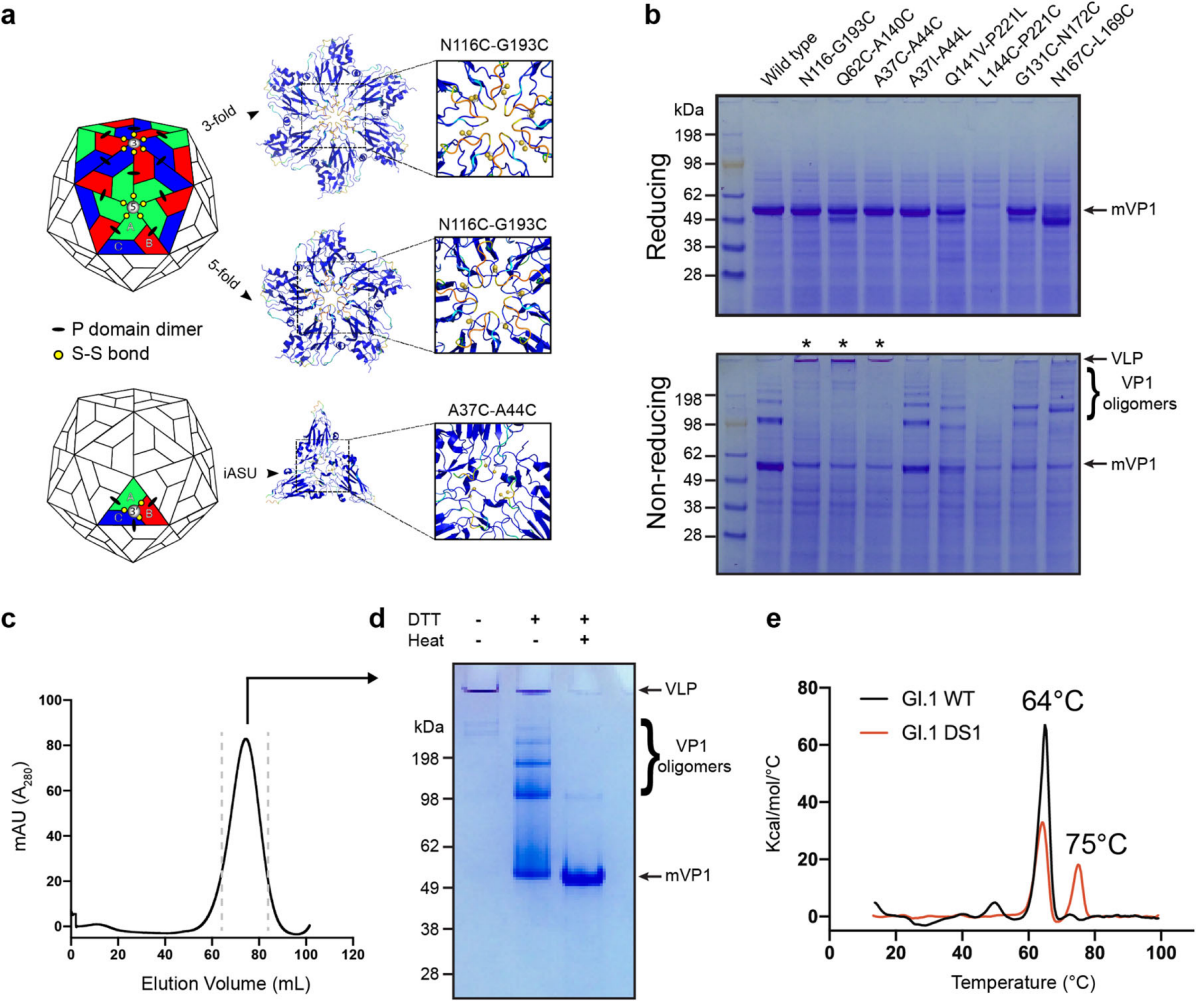
Structure-based design of interprotomer disulfides between VP1 monomers leads to stabilized particles with increased thermal stability of the shell domain. **a** Two strategies can be used to form interprotomer contacts between adjacent VP1 protomers: (1) design of disulfides (or other intermolecular interactions) at the strict fivefold and strict threefold symmetry axes (top panel), and (2) design of disulfides (or other interactions) within the icosahedral asymmetric unit (iASU; bottom panel). One example of each strategy is shown on a portion of GI.1 VLP structure (P domain omitted for clarity). **b** Screening of eight double-point mutants within GI.1 shell domain after oxidation in diamide. Top gel shows the expression levels of VP1 mutants in reducing conditions. Bottom gel shows the same samples in nonreducing conditions. Asterisks indicate mutants that prevent VLP dissociation (intact VLPs fail to enter the separating gel due to their size). Notice the presence of other VP1 oligomers (dimers, tetramers, and larger species). **c** Preparative-scale purification of diamide-treated mutant N116C–G193C (separation performed on Sephacryl S500 column). **d** Stabilization of the final product tested in reducing and nonreducing conditions. Notice that treatment with reducing agent alone does not entirely dissociate particles into VP1 monomers (middle lane). **e** Differential scanning calorimetry thermogram of GI.1 WT and oxidized GI.1 DS1. The peak at 64 °C corresponds to the unfolding of the p domain, while the peak at 75 °C corresponds to the unfolding of the shell domain. (In all gels, mVP1 indicates monomeric VP1 protein).

Disassembly of multivalent antigen particles can decrease immunogenicity^22^ and expose epitopes that would otherwise be inaccessible on the surface of an intact particle. Due to their highly conserved nature, antibodies directed toward these occluded epitopes would tend to dominate the immune response. Norovirus capsids are made of 180 identical copies of the VP1 protein arranged in a *T* = 3 icosahedral particle. The icosahedral asymmetric unit contains three quasi-equivalent VP1 monomers (designated A, B, and C)^23^. Each monomer associates with another monomer to form P domain dimers (in either A–B or C–C configuration). To prevent the disassembly of VLP and exposure of occluded non-neutralizing epitopes, we engineered interprotomer disulfides in the shell domain of GI.1 VLPs. Because of the symmetry of the norovirus VLPs, a cysteine pair introduced near the strict fivefold symmetry axes would yield disulfides between every A–A monomer pairs. The same cysteine pair at the strict threefold symmetry axes would yield disulfide bonds between every B–C monomer pair. (Fig. 2a—top panel). A similar strategy could also be employed to generate disulfides between the A, B, and C monomers of the icosahedral asymmetric unit (Fig. 2a—bottom panel). In both cases, the combination of engineered disulfides and the presence of the P domain dimers between A–B and C–C monomers would result in highly linked VLPs. Starting from the available high-resolution structures of GI.1 VLPs^23,24^, we designed pairs of cysteine mutants that would result in interprotomer disulfides. To evaluate the stability of the different constructs, we used a screening approach developed for the stabilization of the hepatitis B core antigen VLPs^25^. Supernatants from insect cells expressing VP1 were incubated with diamide (to establish an oxidizing environment) and subsequently separated by SDS–PAGE under reducing or nonreducing conditions. VLPs, in which each protomer was covalently linked to the neighboring protomer by disulfide links, did not dissociate in the presence of SDS and thus failed to enter the separating gel due to the large size of the particle (~10 MDa), remaining at the top of the well. In the presence of DTT, the disulfides were reduced, and the particle could be dissociated by SDS, resulting in a single band corresponding to the VP1 monomer. Three constructs showed significant stabilization after oxidation in diamide (Fig. 2b and Supplementary Fig. 4). GI.1 with N116C and G193C mutations (herein referred to as GI.1 DS1) was chosen for large-scale production and characterization (Fig. 2c, d). The resulting particles showed increased thermal stability as evidenced by the appearance of a second melting transition at 75 °C compared to a single transition at 64 °C for the wild-type particles (Fig. 2e). The lower temperature transition corresponds to the unfolding of the P domain, while the higher temperature transition likely corresponds to the stabilized shell domain. These results are in agreement with previous studies on the thermal stability of GI.1 shell domain^26^. We also tested the possibility of introducing hydrophobic interactions (A37I–A44L and Q141V-P221L) to promote stabilization of the VLPs. However, none of the constructs resulted in a stabilizing effect comparable to that obtained with the introduction of cysteine mutations (Fig. 2b).

**Fig. 3:**
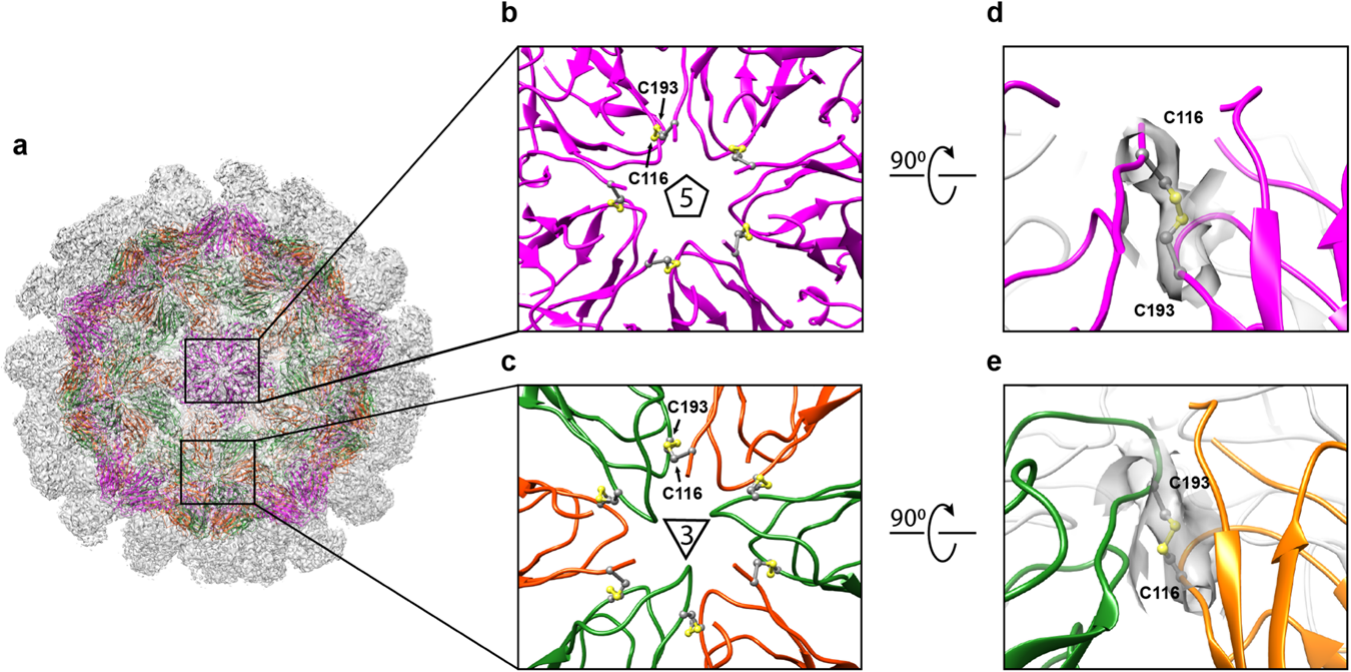
Cryo-EM reconstruction of GI.1 DS1 VLP provides details of interprotomer disulfide bonds between Cys116 and Cys193. **a** Overall electron density of GI.1 DS1 VLPs. Atomic model of the shell is colored according to each nonequivalent monomers. Monomers A, B, and C are shown in magenta, orange, and green, respectively. **b**, **c** Close-up view of the region around **b** the strict fivefold and **c** threefold symmetry axes of the icosahedron. **d**, **e** Details of the electron density for one of the interprotomer disulfide bond between **d** A–A monomers and **e** B–C monomers.

To verify the formation of the disulfides, we determined the structure of the GI.1 DS1 VLP at 3.9 Å resolution using single-particle cryo-electron microscopy (Table 1, Fig. 3, and Supplementary Fig. 3). GI.1 DS1 VLPs showed the characteristic icosahedral symmetry with *T* = 3 arrangement. Clear densities for the formation of the interprotomer disulfide between Cys116 and Cys193 were visible around the fivefold and threefold symmetry axes (Fig. 3b–e). In summary, we successfully designed cysteine mutations in the GI.1 shell domain that resulted in interprotomer disulfides, stabilizing the entire capsid.

**Table 1:** Cryo-EM data collection and refinement statistics.

	GI.1 DS1 (N116C–G193C)
EMDB ID	EMD-22897
PDB ID	7KJP
Data collection	
Microscope	FEI Titan Krios
Voltage (kV)	300
Electron dose (e^−^/Å^2^)	70.48
Detector	Gatan K2 Summit
Pixel Size (Å)	1.073
Defocus Range (µm)	−0.11 to −3.89
Magnification	22,500
Reconstruction	
Software	cryoSparcV2.14
Particles	23,476
Symmetry	Icosahedral
Box size (pix)	600
Resolution (Å) (FSC_0.143_)	3.86
Refinement (Phenix, asymmetric unit)	
Protein residues	515
CC (mask)	0.77
EMRinger Score	2.46
R.m.s. deviations	
Bond lengths (Å)	0.002
Bond angles (°)	0.485
Validation	
Molprobity score	1.43
Clash score	3.54 (4.61^a^)
Rotamer outliers (%)	0.0
Ramachandran	
Favored regions (%)	95.84
Disallowed regions (%)	0

^a^Clash score for full reconstruction of biological unit.

### Stabilized GI.1 VLPs do not expose occluded epitopes but retain antigenicity of blockade epitopes

**Fig. 4:**
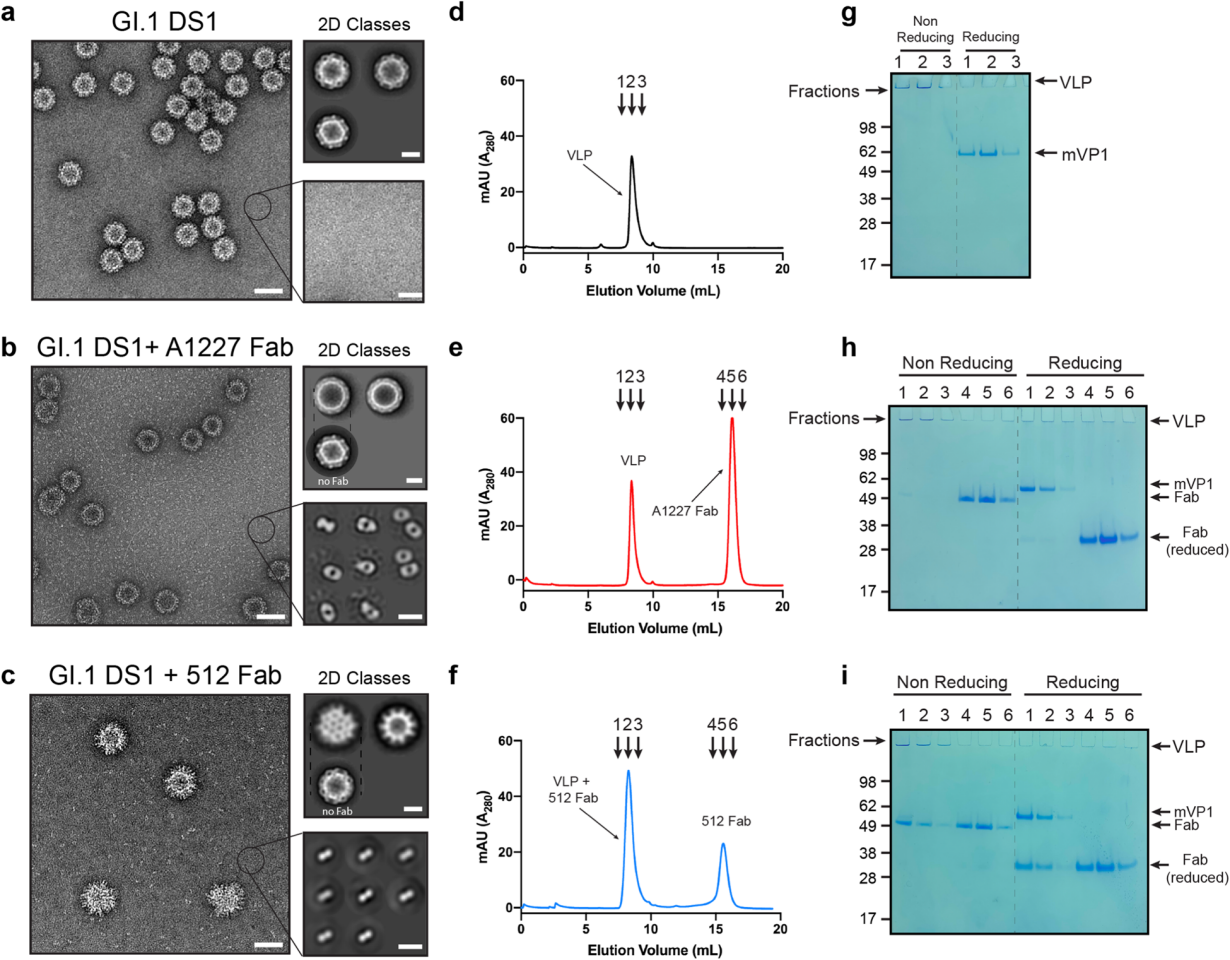
Stabilization of GI.1 VLPs prevents VLPs from disassembling but preserves accessibility to 512 blockade epitope. **a** Representative micrograph of stabilized GI.1 VLPs (GI.1 DS1) in the absence of antibody. Particles appear to be intact, and no disassembled components are visible. 2D class averaging yields very homogeneous particle classes with an approximate diameter of 40 nm (top right panel) and no sign of dissociated molecule (bottom right panel). **b** Representative micrograph of stabilized VLPs after incubation with A1227 Fab. 2D class averages show two species: intact particles with no Fab bound (top right panel) and Fab fragments (bottom right panel). **c** Representative micrograph of stabilized VLPs after incubation with 512 Fab. 2D class averages show two species: intact particles decorated with 512 Fabs (top right panel) and free Fab fragments (bottom right panel). Notice that the sizes of the intact particles increase by ~7 nm, consistent with the presence of a layer of Fabs bound to each particle. **d** The sample in **a** was separated by size-exclusion chromatography and **c** fractions analyzed by SDS–PAGE in reducing and nonreducing conditions. Only one peak is present at the void volume. Nonreducing gel shows only bands at the top of the wells, consistent with the presence of intact stabilized particles. Notice that no dissociated components are detected. **e** Size-exclusion profile of stabilized VLPs incubated with 1A227 Fab and **f** corresponding fractions analyzed by SDS–PAGE. Only two peaks are present: one at the void volume (intact particles) and one corresponding to free Fabs. **h** Size-exclusion profile of stabilized particles incubated with 512 Fab and **i** corresponding fractions analyzed by SDS–PAGE. Only two peaks are present: one at the void volume (intact particles with bound 512 Fabs) and one corresponding to free 512 Fabs. In all gels, mVP1 indicates VP1 monomer. Scale bars are 50, 20, and 10 nm for micrographs, 2D classes of VLPs, and 2D classes of small proteins, respectively.

Stabilization of the VLPs should prevent the binding of occluded-site antibodies, while maintaining accessibility of blockade epitopes. First, NS-EM analysis of GI.1 DS1 revealed the presence of very homogenous VLPs with undetectable amounts of dissociated particles (Fig. 4a). Size-exclusion chromatography (Fig. 4d) and analysis of fractions (Fig. 4g) confirmed the presence of a single species. Critically, the addition of A1227 Fab did not lead to any detectable interaction with the stabilized particles, and because no dissociated VLP components were present in the sample, no complexes could be observed with VP1 dimers (Fig. 4b). From 2D class averages, it appeared that the presence of A1227 Fab did not lead to any significant differences in particle diameter, and the smaller components visible in the background were free Fabs (Fig. 4b). In contrast, addition of 512 Fab to GI.1 DS1 resulted in particles fully decorated with Fabs (Fig. 4c). The diameter of the VLP in complex with the 512 Fab increased by ~7 nm, consistent with a shell of Fabs bound to the surface of the particle. Again, the small objects in the background represented free Fabs (Fig. 4c). Size-exclusion chromatography of VLPs in the presence of Fabs and analysis of fractions by SDS–PAGE confirmed the NS-EM data (Fig. 4e, f, h, i).

To verify that the stabilization of the GI.1 VLPs did not compromise the blockade activity of GI.1-specific antibodies, we performed blockade and binding assays using pig gastric mucin (PGM)^15^ (Supplementary Fig. 5a, d). Binding of both GI.1 WT and GI.1 DS1 was blocked by antibody 512, while occluded-site antibody A1227 did not show any blockade activity (Supplementary Fig. 5b, c). When VLPs were captured on PGM-coated plates, antibody A1227 could only bind to the GI.1 WT VLPs, but negligible binding was observed to GI.1 DS1 (Supplementary Fig. 5e, f). The same experiments were repeated using blockade antibody NVB106 (ref. ^27^) and cross-reactive, but non-blockade antibody A401 (ref. ^17^). Both NVB106 and A401 could bind to wild-type particles (Supplementary Fig. 5g). However, when tested against GI. DS1, only NVB106 showed similar binding, while A401 did not bind (Supplementary Fig. 5h). To further confirm that stabilization prevented binding of A1227 while preserving binding of 512, we used isothermal titration calorimetry (Supplementary Fig. 6). As expected, no binding was detected when A1227 Fab was titrated into GI.1 DS1 VLPs (Supplementary Fig. 6a), while 512 Fab bound with high affinity to the particles. Analysis of the ITC data showed that ~100 512 Fabs were bound to each particle. (Supplementary Fig. 6b). Overall, our data showed that stabilizing GI.1 VLPs by engineering interprotomer disulfide bonds in the shell domain prevented dissociation of the particles, but retained antigenicity of blockade epitopes (512 and NVB106). In addition, non-neutralizing antibodies (A1227 and A401) were no longer capable of binding the intact particles.

### Stabilized VLPs elicit blockade antibodies faster than wild type and focus immune responses toward blockade epitopes

Current clinical trials using norovirus VLPs have proven partially successful. The bivalent GI.1/GII4 VLP vaccine, currently in phase IIb clinical trials, is highly immunogenic; however, blockade titers wane very quickly, and for some individuals boosting fails to increase blockade responses^15^. We reasoned that lack of particle stability could promote exposure of immunodominant epitopes (mostly located at the base of the P domain and within the shell region). The resulting antibodies (targeting these occluded sites) would appear cross-reactive, but would fail to neutralize the virus. Using stabilized particles should allow greater availability of intact particles to B cells in vivo for elicitation of higher blockade titers. In addition, since the stabilized particles would not present occluded-site epitopes, they could help focus the immune response toward accessible (and potentially neutralizing) epitopes.

**Fig. 5:**
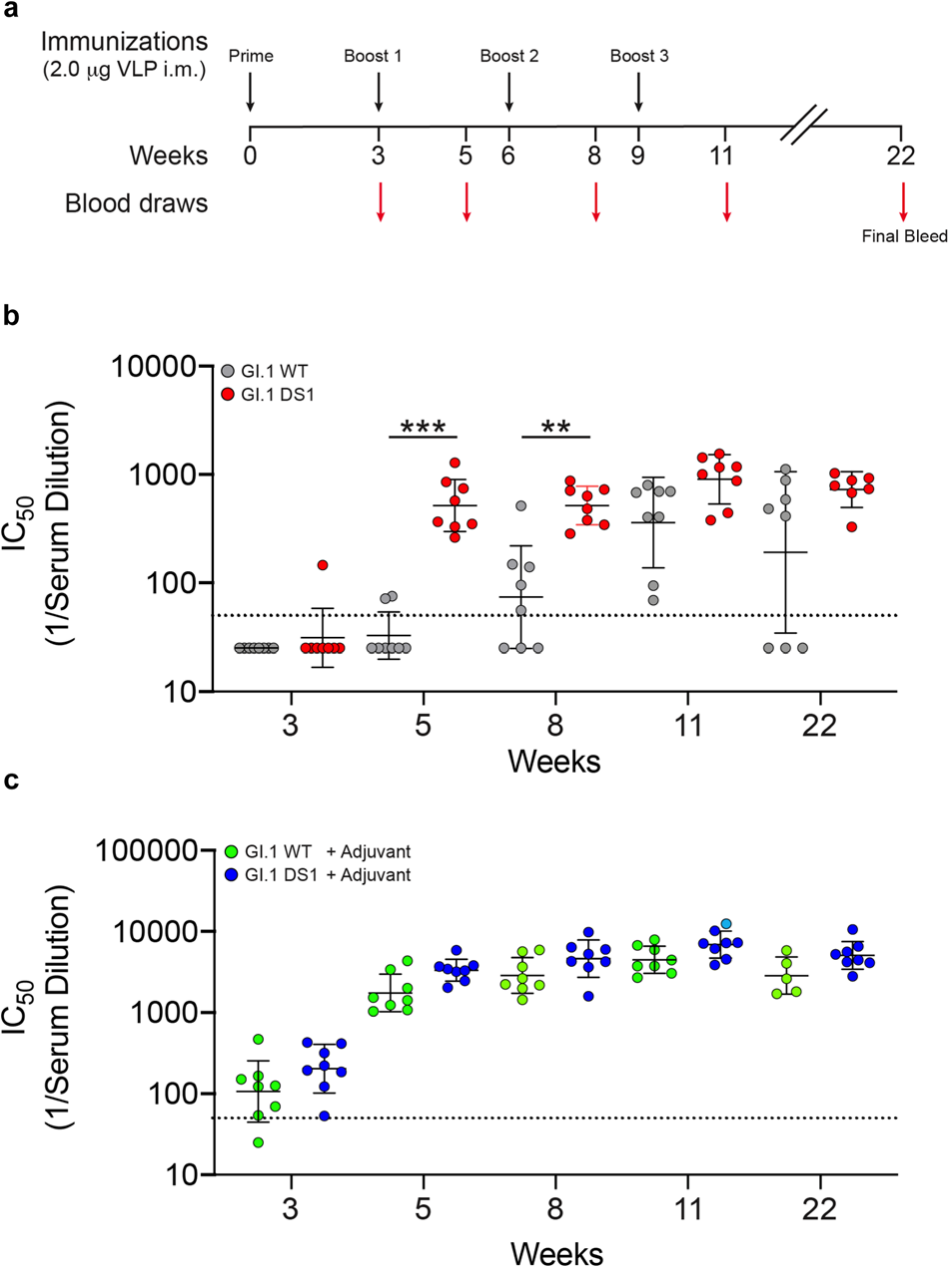
Mice immunizations with stabilized GI.1 VLPs produces high titers of blockade antibody responses after two injections without alum. **a** Balb/c mice were immunized with two micrograms of either GI.1 WT or GI.1 DS1 VLPs intramuscularly at weeks 0, 3, 6, and 9. Blood draws were performed at weeks 3, 5, 8, and 11. To test durability of immune response, final bleed was performed 13 weeks after the last immunization. For each blood draw, HBGA blockade titers were assessed. **b** Comparison of blockade antibody titers between GI.1 WT (gray circles) and GI.1 DS1 (red circles) in the absence of adjuvant. Disulfide stabilization of norovirus VLPs leads to fast development of blockade titers compared to wild-type VLPs (compare blockade titers after second immunization). **c** Comparison of blockade titers between GI.1 WT (green circles) and GI.1 DS1 (blue circles) in the presence of adjuvant (alum). No significant differences at any time point are observed, indicative of potentially stabilizing effect of GI.1 WT VLPs by alum. Limit of detection (titer = 80) is indicated with a horizontal dotted line. Geometric mean titers ± geometric SD are shown in scatter dot plot. *P* values were determined by two-tailed Mann–Whitney tests. *P ≤ 0.05, **P ≤ 0.01, ***P ≤ 0.001. There were eight mice per group for VLP immunizations and six mice per group for controls (PBS and PBS + adjuvant).

To test our hypotheses, we immunized mice with GI.1 WT and GI.1 DS1 VLPs. Each group was tested with and without adjuvant (alum). The first boost was administered three weeks after the prime, followed by a second and third boost at weeks 6 and 9, respectively. Blood draws were performed at weeks 3, 5, 8, and 11, and final bleed was performed at week 22 (Fig. 5a). Blockade assays were performed after each blood draw (Fig. 5b, c and Supplementary Fig. 7). In the absence of alum, mice immunized with the stabilized particles showed high blockade titers after only two immunizations, but the titers did not increase significantly with subsequent boosts (Fig. 5b—red circles). In contrast, blockade titers using GI.1 wild-type particles were undetectable until the third immunization was administered. Even after four immunizations with GI.1 WT particles, some animals showed barely detectable blockade titer (Fig. 5b—gray circles). However, when VLPs were administered with adjuvant, we did not see any significant difference at any time point between stabilized and wild-type particles (Fig. 5c), suggesting a stabilizing effect of adjuvant on the wild-type particles.

**Fig. 6:**
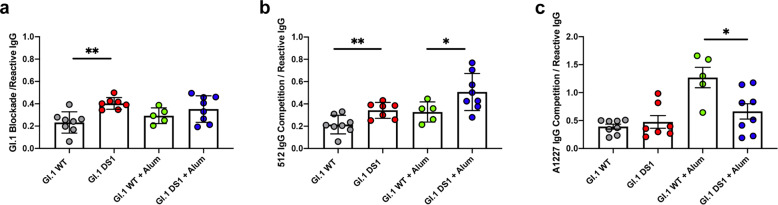
Stabilization of GI.1 VLPs focuses immune responses toward blockade epitopes and away from occluded epitopes. **a** Ratio of blockade titers over total GI.1-reactive IgG titers at week 22. In the absence of alum, immunization with GI.1 DS1 leads to a twofold increase in blockade titers relative to total GI.1-reactive titers, compared to immunization with GI.1 WT. **b** Ability of serum antibodies at week 22 to compete with blockade antibody 512, relative to total GI.1-reactive titers. For both non-adjuvanted and adjuvanted groups, sera from mice immunized with stabilized VLP can compete with 512 binding more than sera from mice immunized with wild-type VLPs. **c** Ability of serum antibodies at week 22 to compete with occluded-site antibody A1227, relative to total GI.1-reactive titers. In the presence of alum, sera from mice immunized with wild-type VLPs can compete with A1227 more than sera from immunization with stabilized VLPs. Mean ± SD are shown in each box plot. *P* values were determined by two-tailed Mann–Whitney tests. *P ≤ 0.05, **P ≤ 0.01.

Stabilized GI.1 VLPs should elicit higher titers of blockade antibodies (relative to GI.1-reactive antibody titers) than GI.1 WT. Indeed, immunization with stabilized particles led to a twofold increase of blockade antibodies relative to total binding responses in the absence of alum (Fig. 6a and Supplementary Fig. 8a). In addition, when sera from immunized mice were used in a competition assay with the blockade antibody 512 for binding to the VLPs, we observed more competition for sera from stabilized particles compared to sera from wild-type particles, when normalized to total GI.1-binding titers (Fig. 6b and Supplementary Fig. 8a, b). The same experiment was repeated using the occluded-site antibody A1227. At least for the adjuvanted groups, the sera from mice immunized with wild-type particle were able to compete with the A1227 binding to VLPs more than sera from mice immunized with stabilized VLPs (Fig. 6c and Supplementary Fig. 7a–c). As an orthogonal approach, we used biolayer interferometry to measure the residual serum binding to VP1 dimers after complex formation with either 512 or A1227 antibodies (Supplementary Fig. 9a, b). VP1 dimers were purified from dissociated GI.1 VLP samples (Fig. 1d). After capturing VP1 dimers with 512-IgG, sera from mice immunized with stabilized particles showed significantly reduced residual binding to the dimer, compared to sera from wild-type immunization at the same dilution (Supplementary Fig. 9c). Although not statistically significant, the results from the same experiment using A1227 bound to VP1 dimers showed an opposite trend. Sera from mice immunized with wild-type particles had less residual binding to the VP1/A1227 complex, while sera from mice immunized with stabilized VLPs had a higher residual binding (Supplementary Fig. 9d). In the presence of alum, sera from mice immunized with GI.1 WT and GI.1 DS1 had similar residual binding to VP1 dimers (Supplementary Fig. 9e, f).

Taken together, these data support our hypothesis that preventing norovirus particles from dissociating can lead to antibody responses that are more focused toward blockade epitopes and, at least in the absence of alum, the stabilized VLPs showed faster development of blockade titers compared to wild-type VLPs.

## Discussion

In this study, we found that purification of GI.1 wild-type VLPs consistently resulted in a mixture of intact particles and partially dissociated VLP components. Even after extensive purification with size-exclusion chromatography, it was still possible to detect disassembled particle components. NS-EM analysis of these smaller components revealed that the primary species were VP1 dimers. Interestingly, the cross-reactive but non-neutralizing antibody A1227 (Fab fragment) bound exclusively to VP1 dimers, while no Fab could be detected on the surface of intact particles. Conversely, the blocking antibody 512 could be found in complex with both intact particles and VP1 dimers. This suggests a potential route for the elicitation of cross-reactive but non-neutralizing antibodies, previously isolated from humans and mice immunized with norovirus VLPs^17–19^. When animals are immunized with VLPs, the partially dissociated particles expose highly conserved sites (mostly located at the base of the P domain and the shell domain), leading to the elicitation of antibodies capable of binding dissociated particles from several genogroups. It has been shown that cross-reactive but non-neutralizing antibodies are also elicited in people infected with GI.1 viruses, indicating that even infectious noroviruses could present occluded epitopes^27^. It is currently unclear if noroviruses can naturally present occluded sites resulting from assembly defects or metastability of the capsid. It has been suggested that the flexibility of the P domain could allow some of these occluded epitopes to be (at least transiently) exposed^28,29^. On the other hand, to date, there are no EM studies showing direct evidence of VLPs bound to occluded-site antibodies. In our experiments, we could not detect any A1227 Fab bound to intact particles by NS-EM. However, we cannot exclude that binding of A1227 Fab at very low occupancy (few Fabs per VLP) could happen due to the transient exposure of occluded epitopes. The resolution of negatively stained samples is not sufficient to detect few Fabs that bind at the base of the P domain. Although infectious norovirus particles appear to be in a *T* = 3 icosahedral configuration^30^, there is evidence that heterologous expression of VP1 proteins can lead to the assembly of *T* = 1, *T* = 3, and *T* = 4 icosahedral VLPs^24,30^, underscoring how experimental procedures can have a substantial impact on the final morphology of the particles. In light of these discrepancies, it will be valuable to investigate the structural features of intact virions and their interactions with antibodies and compare them to heterologously expressed VLPs.

To prevent antibody responses to occluded sites, we followed a stabilization approach previously used to stabilize HBcAg and Qβ particles^25,31^, as well as foot-and-mouth disease viruses^32,33^. The design of cysteine pairs within the shell domain of VP1 monomers can lead to the formation of intermolecular disulfide bonds between A–A and A–B monomers. This, in turn prevents the disassembly of the particle into VP1 dimers. A similar approach has recently been used to stabilize a chimera composed of norovirus GII.4 shell domain fused to the rotavirus VP8. In that case, however, the protein self-assembled into *T* = 1 icosahedral VLPs, and the disulfide bonds could not be visualized due to the low resolution of the EM reconstructions^34^.

In addition to preventing disassembly, stabilization of VLPs also impacted immunogenicity. In particular, stabilized VLPs elicited a more focused immune response toward accessible epitopes, and (in the absence of adjuvant) the development of blockade titers was much faster than immunization with wild-type particles. Interestingly, the presence of adjuvant drastically improved the immunogenicity of wild-type particles. We speculate that adsorption of VLPs on adjuvant could exert a stabilizing effect, thereby alleviating their propensity to dissociate. Overall our data point to a potential avenue for the improvement of current GI.1 VLP vaccines. Furthermore, it will be interesting to evaluate the effect of particle stabilization in the context of other human norovirus genogroups and genotypes, such as the medically important GII.4 or emerging strains, such as GII.2 and GII.6. Finally, the ability of stabilized norovirus VLPs to discriminate between antibodies binding to irrelevant epitopes and surface-exposed epitopes can be exploited in antibody isolation campaigns to search for broadly neutralizing antibodies.

## Methods

### Production of norovirus virus-like particles

The gene for GI.1 VP1 protein (accession number: Q83884) was synthesized, codon optimized for insect cell expression, and cloned in a pFastBac1 vector (GenScript). Mutations were performed by GeneImmune using the original pFastBac vector. All plasmids were sequenced before use. Generation of recombinant bacmid DNA was done using the Bac-to-Bac Baculovirus Expression System according to manufacturer instructions (Invitrogen).

Sf9 cells were maintained in ESF921 medium (Expression systems) and transfected with recombinant bacmid DNA, using a mixture of 1 µg of DNA and 8 µL of Cellfectin II (Invitrogen) in a final volume of 100 µL. After incubation for 1 h at room temperature, 800 µL of transfection reagent (Expression system) was added. Transfection was carried out in six-well plates containing a total of 0.9 × 10^6^ cells per well by dropwise addition of transfection mixture. After incubation for 4 h at 27 °C, the medium was removed, and 3 mL of ESF931 medium was added. Cells were incubated at 27 °C for 6 days. Cell culture medium containing recombinant baculovirus (P1 generation) was collected from each well and filter sterilized through 0.2 µm filters. High titer baculovirus was obtained by infecting 50 mL Sf9 cells at a density of 1 × 10^6^ cell/mL with 0.5 mL of P1 virus and incubating for 6 days (27 °C, 140 r.p.m.). Medium containing baculovirus (P2 generation) was subsequently clarified by centrifugation (4000 × *g*, 45 min, 4 °C) and filter sterilized through 0.2 µm filters and kept at 4 °C protected from light until needed. To test expression of VP1 proteins, 50 mL of Sf9 at 3 × 10^6^ cells/mL were infected with 5 mL of P2 virus and incubated for 4 days (27 °C, 140 r.p.m.) before clarification of medium. Expression levels were assessed by SDS–PAGE of samples from clarified medium.

Large-scale preparation of VLPs was carried out in 200 mL of SF9 cells at 3 × 10^6^ cells/mL by addition of baculovirus at MOI of 1:5 (Sf9:PFU) for 4 days at 27 °C. Clarified supernatant was prepared as described above. VLPs were concentrated by centrifugation (54,000 × *g* for 2 h at 4 °C) on a cushion of 3 mL of 60% iodixanol (Optiprep). Most of the content of the tube was removed by pipetting, leaving the bottom 3 mL, the concentrated protein layer, and an additional 3 mL above the layer. This resulted in a final iodixanol concentration in the sample of 30%. The mixture was transferred to 5.5 mL Quick-Seal® Ultra-Clear tubes (Beckmann) and centrifuged at 300,000 × *g* for 8 h at 4 °C in a NVT100 rotor. The clearly visible VLP layer was collected by side puncture and injected onto a 16/60 Sephacryl S-500 gel filtration column equilibrated with phosphate-buffered saline (PBS). The VLP peak eluted at ~74 mL, and fractions were pooled, concentrated to ~1 mg/mL in Amicon Ultra Filters (MWCO 30 kDa), and stored at 4 °C until needed. In the case of stabilized mutants, the pooled VLP peak was incubated with a final concentration of 20 mM diamide for 1 h at room temperature, and subsequently dialyzed overnight against PBS or reinjected onto Sephacryl S-500 columns to remove free diamide. Confirmation of disulfide formation was assessed by SDS–PAGE, with samples run in reducing and nonreducing conditions.

### Production of antibodies

Antibodies and Fab fragments were produced as previously described^17^. Briefly, heavy and light chain plasmids (IgG format) containing secretion signals were co-transfected in Expi293F cells (ThermoFisher) using Turbo293 transfection reagent (Speed Biosystem). Cells were incubated for 1 day at 37 °C, followed by 4 days at 37 °C. All subsequent steps were performed at 4 °C. Supernatant was collected by centrifugation and loaded onto Protein A resin (GE Healthcare) pre-equilibrated with PBS. Bound antibodies were washed with 50 ml of PBS and eluted dropwise in 1 mL fractions with Pierce IgG Elution buffer (Pierce). Elution was neutralized with 1 M Tris-Cl, pH 8.0 (final concentration 0.1 M). Fractions with highest A_280_ absorption were pooled and dialyzed overnight against PBS. Dialyzed protein was concentrated to ~10 mg/mL, filter sterilized, and kept at 4 °C until needed. For the production of Fab fragment, the purified antibodies were incubated with HRV-3C protease (Millipore-Sigma) overnight at 4 °C. Cleavage reaction was loaded onto Protein A resin (GE Healthcare), and flow-through was collected. Fabs were purified by size-exclusion chromatography on a Superdex 200 16/60 column in PBS. Fractions corresponding to Fab were pooled, concentrated to ~5 mg/mL, filter sterilized, and kept at 4 °C until needed.

### Negative staining electron microscopy

VLP samples were diluted to ~0.1 mg/mL with 10 mM HEPES, pH 7.0, and 150 mM NaCl. Higher dilutions, in the range of 0.01–0.05 mg/mL, were used when dissociated VLP fragments or Fab fragments were present. Material was adsorbed to a glow-discharged carbon-coated copper grid, washed with the same buffer, and negatively stained with 0.75% uranyl formate. Datasets were collected at magnifications of 50,000 and 100,000 (pixel size: 0.44 and 0.22 nm, respectively) using SerialEM^35^ on an FEI Tecnai T20 electron microscope equipped with a 2k × 2k Eagle CCD camera and operated at 200 kV, as well as at a magnification of 57,000 (pixel size: 0.25 nm) using EPU on a ThermoFisher Talos F200C electron microscope equipped with a ThermoFisher Ceta CCD camera and operated at 200 kV. Particles were picked automatically using in-house developed automatic software (unpublished) or using e2boxer from the EMAN2 software package^36^, followed by manual correction. Reference-free 2D classifications and 3D reconstructions were performed using Relion^37^.

### Analytical size-exclusion chromatography to evaluate dissociated VP1 components

Norovirus VLPs (200 µg) were incubated with either 512 Fab or A1227 Fab to a final molar ratio of 1:2 (VP1:Fab) on ice for 1 h. Mixture was subsequently injected onto a Supredex 200 Increase 10/300 GL connected to an Äkta Pure system (GE Healthcare) equilibrated in PBS. Fractions (0.5 mL each) were collected and 20 µL from each fraction was mixed with 20 µL of 2× sample buffer (with and without reduction agent). Fifteen microliters of each fraction were loaded onto a NuPAGE™ 4–12%, Bis-Tris gel, which was subsequently stained with Coomassie. Each gel was derived from the same experiment and was processed in parallel. Uncropped images of the original gels are presented in Supplementary Fig. 10. Integration of peaks from chromatograms was performed with the Evaluation option in the Unicorn 7.3 software.

### Differential scanning calorimetry

VLP samples were prepared by diluting stock solutions (at 2 mg/mL) in PBS to a final concentration of 300 µg/mL. Four hundred microliter of diluted VLPs were loaded on a 96-well plate next to PBS only samples, and heat capacities were measured using a high-precision differential scanning VP-DSC microcalorimeter (GE Healthcare/MicroCal). The scan rate was set at 1 °C per minute from 18 to 110 °C.

### Isothermal titration calorimetry

Binding experiments by ITC were performed at 25 °C using a VP-ITC microcalorimeter from MicroCal-Malvern Instruments (Northampton, MA, USA). GI.1 VLP and the 512 and 1227 antibody fragments were prepared and dialyzed against PBS, pH 7.4. In each titration, the solution containing the antibody fragment was added stepwise in 10 µL aliquots to the stirred calorimetric cell (*v* ~1.4 mL) containing GI.1 DS1 VLP at 12–17 nM. The concentration of Fab in the syringe was 16–27 µM. All reagents were thoroughly degassed prior to the experiments. The results are expressed per mole of Fab fragment and the stoichiometry, *N*, denotes the number of binding sites per mole of VLP. The heat evolved upon each injection was obtained from the integral of the calorimetric signal, and the heat associated with binding was obtained after subtraction of the heat of dilution. The enthalpy change, Δ*H*, the association constant, *K*_a_ (the dissociation constant, *K*_d_ = 1/*K*_a_) and the stoichiometry, *N*, were obtained by nonlinear regression of the data to a single-site binding model using Origin with a fitting function made in-house. Gibbs energy, Δ*G*, was calculated from the binding affinity using Δ*G* = −*RT* ln*K*_a_, (*R* = 1.987 cal/(K × mol)) and *T* is the absolute temperature in kelvin). The entropy contribution to Gibbs energy, *−T*Δ*S*, was calculated from the relation Δ*G* = Δ*H* − *T*Δ*S*.

### Determination of GI.1 DS1 VLP structure by cryo-EM

#### Cryo-EM data collection and processing

GI.1 DS1 was deposited on a C-flat grid 1.2/1.3 (protochip.com) with 2.3 μL of volume at 1 mg/mL concentration. The grid was vitrified with an FEI Vitrobot Mark IV with a wait time of 30 s, blot time of 3 s, and a blot force of 1. Data collection was performed on a Titan Krios microscope using Leginon software^38^. The camera was a Gatan K2 Summit direct detection device. High magnification exposures were collected in movie mode for 10 s with a total dose of 70.48 e^-^/Å^2^ fractionated over 50 raw frames. Images were initially processed using Appion^39,40^; frames were aligned using MotionCor2 (ref. ^41^). CTFFind4 (refs. ^42,43^) was used to calculate the CTF, and DoG Picker^39,40^ was used for initial particle picking. RELION^37^ was then used for particle extraction, and the particle stack was imported to cryoSPARC. CryoSPARC 2.12 (ref. ^44^) was used for 2D classifications, ab initio 3D reconstruction in C1, and the volume was subjected to homogeneous refinement using I1 symmetry.

#### Model building

UCSF Chimera^45^ was used to fit the asymmetric unit of human norovirus GI.1 Norwalk VLP (PDB 6OUT) into cryo-EM density and determine symmetry operators. Residues corresponding to the P domain were excluded from the model because the resolution in this region was insufficient for model building, leaving residues 51–222 of chains A–C. The model was subjected to alternating rounds of real space refinement in Phenix^46^ and manual building in Coot^47^.

Several loops located around the threefold symmetry axis were deleted because of weak density in this region. The FSC curve between the map and the model was calculated using phenix.mtriage. Model validation was performed with MolProbity^48^ and EMRinger^49^. Figures were generated in UCSF Chimera and PyMOL (www.pymol.org).

### EIA and blockade, and competition assays

EIA and blockade assays were done as previously reported^17^. For VLP capture assays, plates were coated with PGM as for blockade assays, followed by addition of 0.25 µg/mL VLP for 1 h at 37 °C and bound mAb detected as for EIA.

The competition between mouse polyclonal serum and human monoclonal antibodies for binding to immobilized VLPs (0.25 μg/mL) was measured by EIA, as described previously^50^. Briefly, mouse sera were added to VLP-coated plates at different dilutions. After 1 h, human mAbs were added at a concentration required to achieve 50% maximal binding [EC_50_] at room 37 C for 1 h. The plates were then washed with PBS–0.05% Tween 20, and bound human mAb was detected using anti-human-IgG HRP (GE Healthcare). The concentration of sera that blocked binding of 50% of the mab was determined as described above for blockade of binding assays.

### Mouse immunization

Mouse studies were executed in accordance with the recommendations for the care and use of animals by the Office of Laboratory Animal Welfare (OLAW) at NIH. The Institutional Animal Care and Use Committee (IACUC) at UNC-CH approved the animal studies performed here (protocol, IACUC 17-059). Six-week-old Balb/c mice (Jackson Labs) were immunized intramuscularly with 2 µg of VLP plus either PBS or 50 µg alhydrogel (Invivogen). Identical booster vaccinations were performed at weeks 3, 6, and 9 with bleeds at weeks 3, 5, 8, and 11. Mice were euthanized with isoflurane at week 22, for terminal bleed. Each VLP group contained eight mice, while six mice were used in PBS control groups.

### Statistical analysis

Statistical analyses were performed using two-tailed Mann–Whitney tests with GraphPad Prism 8.0 software (La Jolla, CA). Differences were considered statistically significant at *P* < 0.05.

### Biolayer interferometry

A fortéBio Octet Red384 instrument was used to measure binding of sera from immunized mice after capture of VP1 dimer with 512 or A1227 IgG. All assays were performed at 1000 r.p.m. agitation. Assays were performed at 30 °C in tilted black 384-well plates (Geiger Bio-One) with final volumes of 50 μL/well. Anti-human-Fc sensor tips were used to capture either 512 or A1228 IgG. Biosensor tips were equilibrated for 30 min in PBS before each experiment. Capture levels were between 1.2 and 1.3 nm, and variability for each tip did not exceed 0.1 nm. Biosensor tips were then equilibrated for 300 s in PBS before a second association step (600 s) with VP1 dimers at 30ug/mL. Biosensor tips were then equilibrated for 300 s in PBS prior to measuring association to serum samples from final bleed (300 s). All sera were diluted 50-fold in PBS. Initial slopes were determined by linear regression of the signal in the first 30 s of association.

### Reporting summary

Further information on research design is available in the Nature Research Reporting Summary linked to this article.

## Supplementary information

Supplementary Information

Reporting Summary

## Data Availability

Cryo-EM maps have been deposited in the Electron Microscopy Data Bank (EMDB ID: EMD-22897), and in the Protein Data Bank (PDB ID: 7KJP). Plasmids used in this study have been deposited in Addgene with the following deposition numbers: ID 162579 (GI.1_WT), ID 162580 (GI.1_N116C-G193C), ID 162581 (GI.1_A62C-A140C), ID 162582 (GI.1_A37A-A44C), ID 162583 (GI.1_A37I-A44L), ID 162584 (GI.1_Q141V-P221L), ID 162585 (GI.1_L144C-P221C), ID 162586 (GI.1_G131C-N172C), and ID 162587 (GI.1_N167C-L169C). All other data from the current study are available from the corresponding authors upon request.
