# Supplementary material for: Disulfide stabilization of human norovirus GI.1 virus-like particles focuses immune response toward blockade epitopes
Source: NPJ Vaccines. 2020 Dec 14;5:110. doi: 10.1038/s41541-020-00260-w (PMC7736355; doi:10.1038/s41541-020-00260-w)
Supplement: Supplementary file 1 — Supplementary Information [file 41541_2020_260_MOESM1_ESM.pdf]

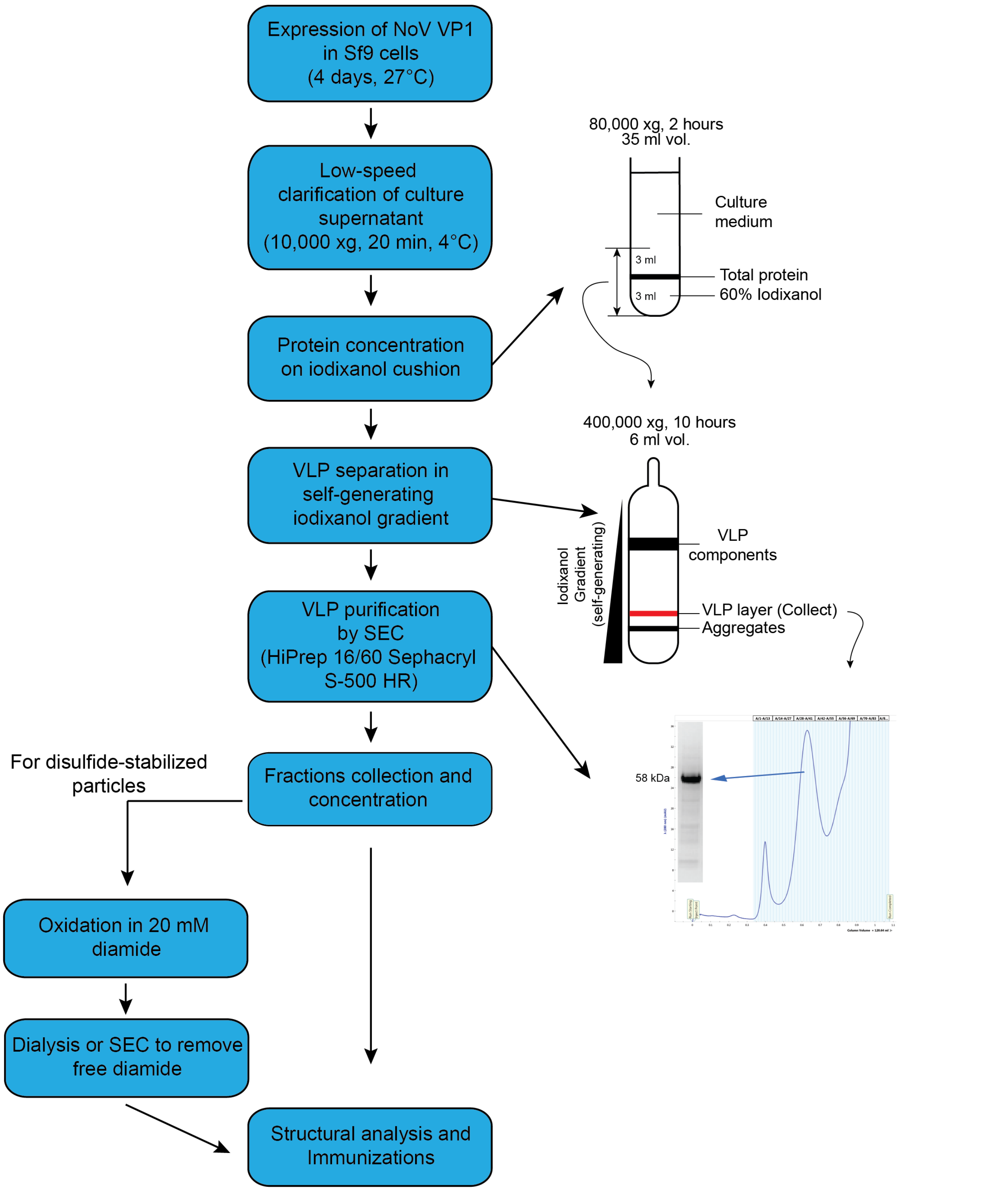

Supplementary Figure 1. Flow-chart for the production of GI.1 VLPs. Particles were expressed in Sf9 cells and collected from the cell culture supernatant. Iodixanol (Optiperp®) was used to concentrate and separate particles by ultracentrifugation. VLP layer was collected by side puncture and injected onto a Sephacryl S500 column. VLP peak eluted around 74mL. Fractions were collected and concentrated in Amicon® spin column (50 kDa MWCO). To promote the formation of disulfide bonds, particles containing cysteine mutations were incubated with diamide for one hour and diamide removed by dialysis or by a second round of size-exclusion chromatography. Concentrated VLPs were used for EM analysis and mice immunizations.

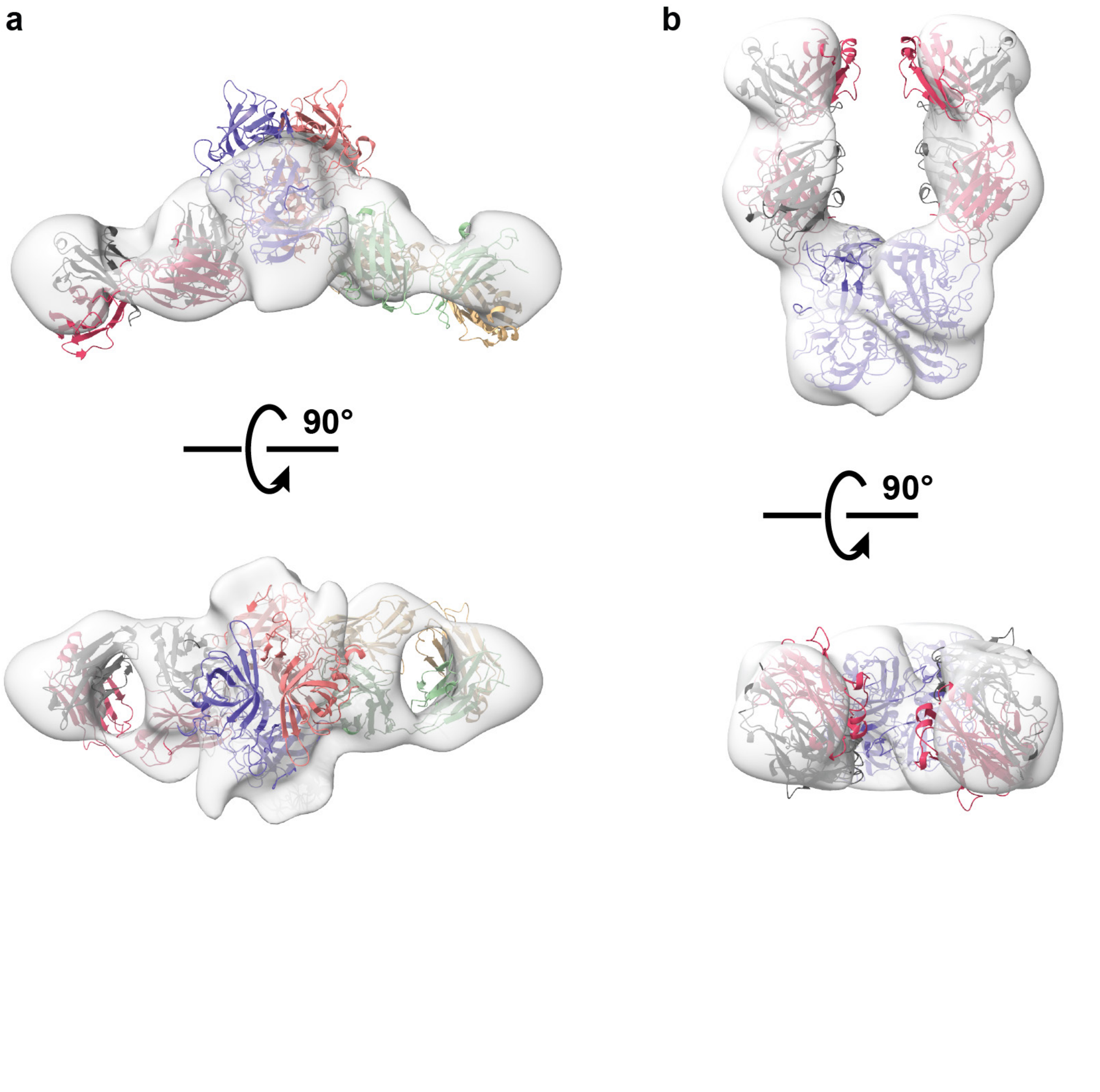

Supplementary Figure 2. Three-dimensional reconstructions of VP1/A1227 Fab complex and VP1/512 Fab complex. (a) Fitting of the X-ray crystal structures of GII.4c P domain in complex with the Fab fragment of antibody A1227 (PDB ID: 6N81) into 3D-reconstruction from negatively stained samples of mixed GI.1 WT VLPs and A1227 Fab. (b) Fitting of the X-ray crystal structures of GI.1 P domain in complex with the Fab fragment of antibody 512 IgA (PDB ID: 5KW9) into 3D-reconstruction from negatively stained samples of mixed GI.1 WT VLPs and 512 Fab.

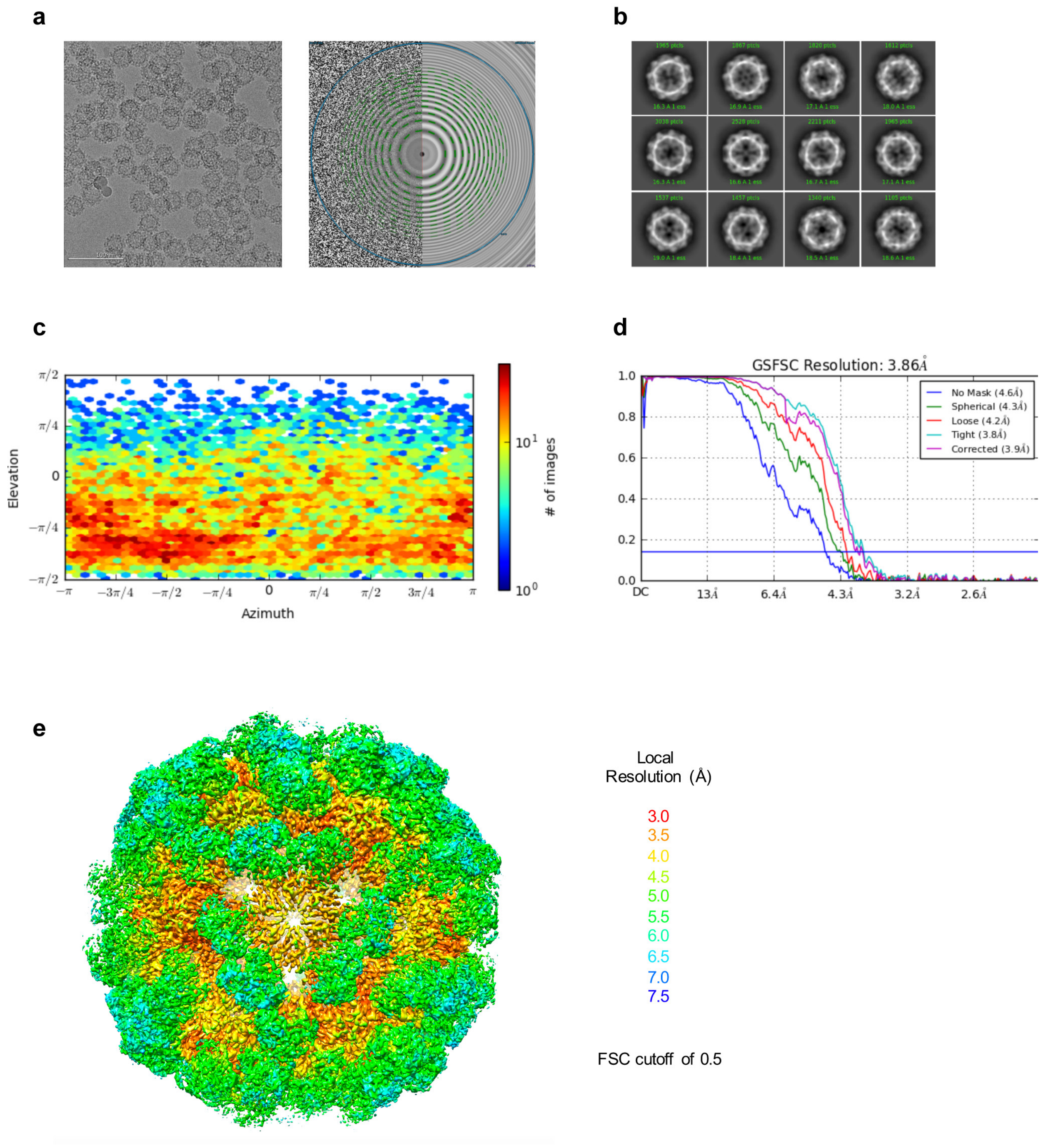

Supplementary Figure 3. Cryo-EM data collection and map refinement. (a) Representative micrograph and CTF of the micrograph are shown. (b) Representative 2D class averages are shown. (c) The orientations of all particles used in the final refinement are shown as a heatmap. (d) The gold-standard Fourier shell correlation resulted in a resolution of 3.86 Å with I symmetry. The horizontal line indicates the 0.143 cutoff threshold (e) The local resolution of the full map is shown generated through cryoSPARC using an FSC cutoff of 0.5.

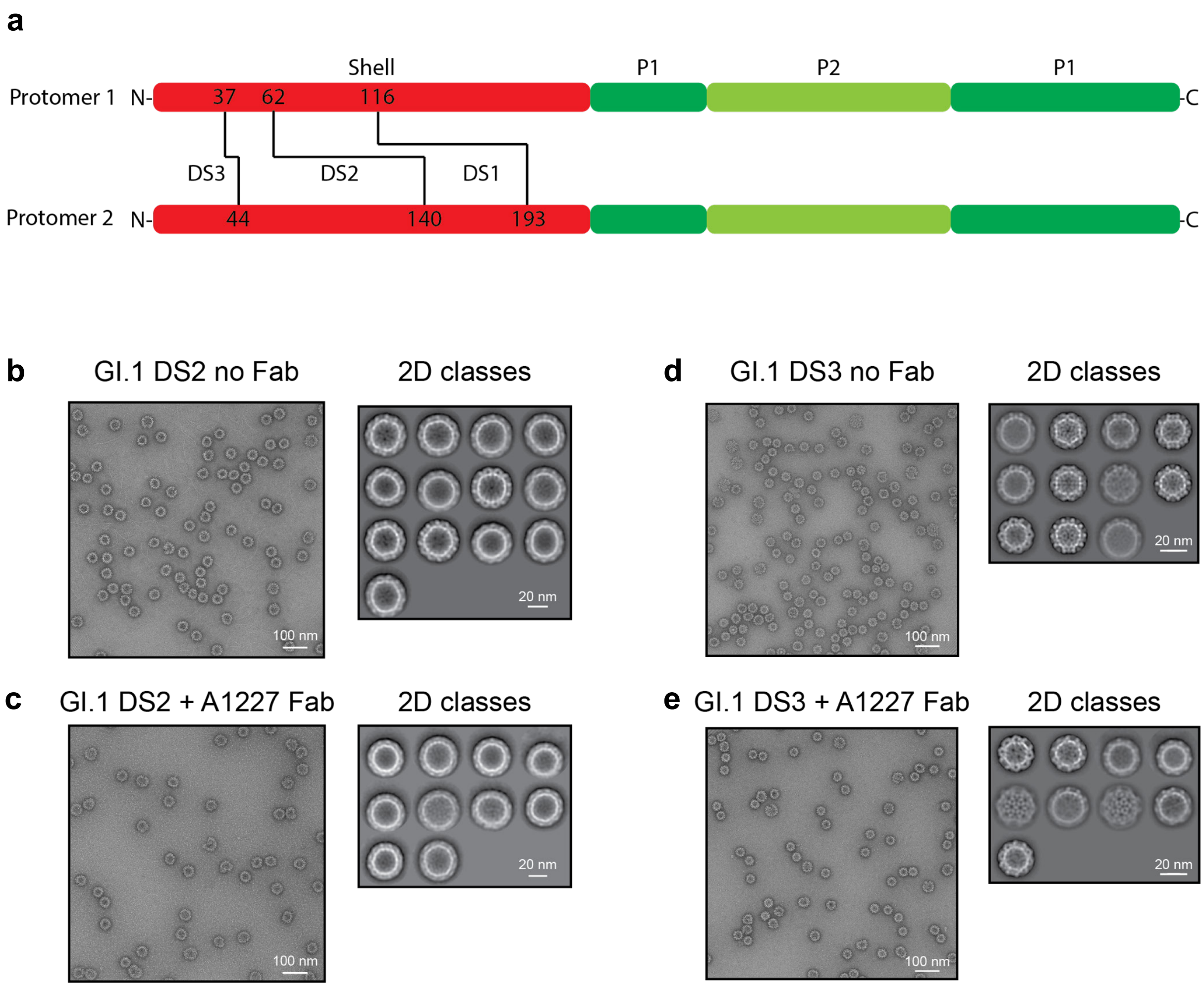

Supplementary Figure 4. Schematic of interprotomer disulfide constructs used to stabilized GI.1 VLPs. (a) Shell domain sequence is shown in red (residues 1 to 225). Predicted disulfide bonds are shown with black lines connecting the cysteines in adjacent protomers. (b) Negative staining representative image of GI.1 DS2 in the absence of Fabs. All particles appear intact and with the correct size. (c) Addition of A1227 Fab did not lead to any changes to the appearance of the particles. (d) Negative staining representative image of GI.1 DS3 in the absence of Fabs. All particles appear intact and with the correct size. (e) Addition of A1227 Fab did not lead to any changes to the appearance of the particles.

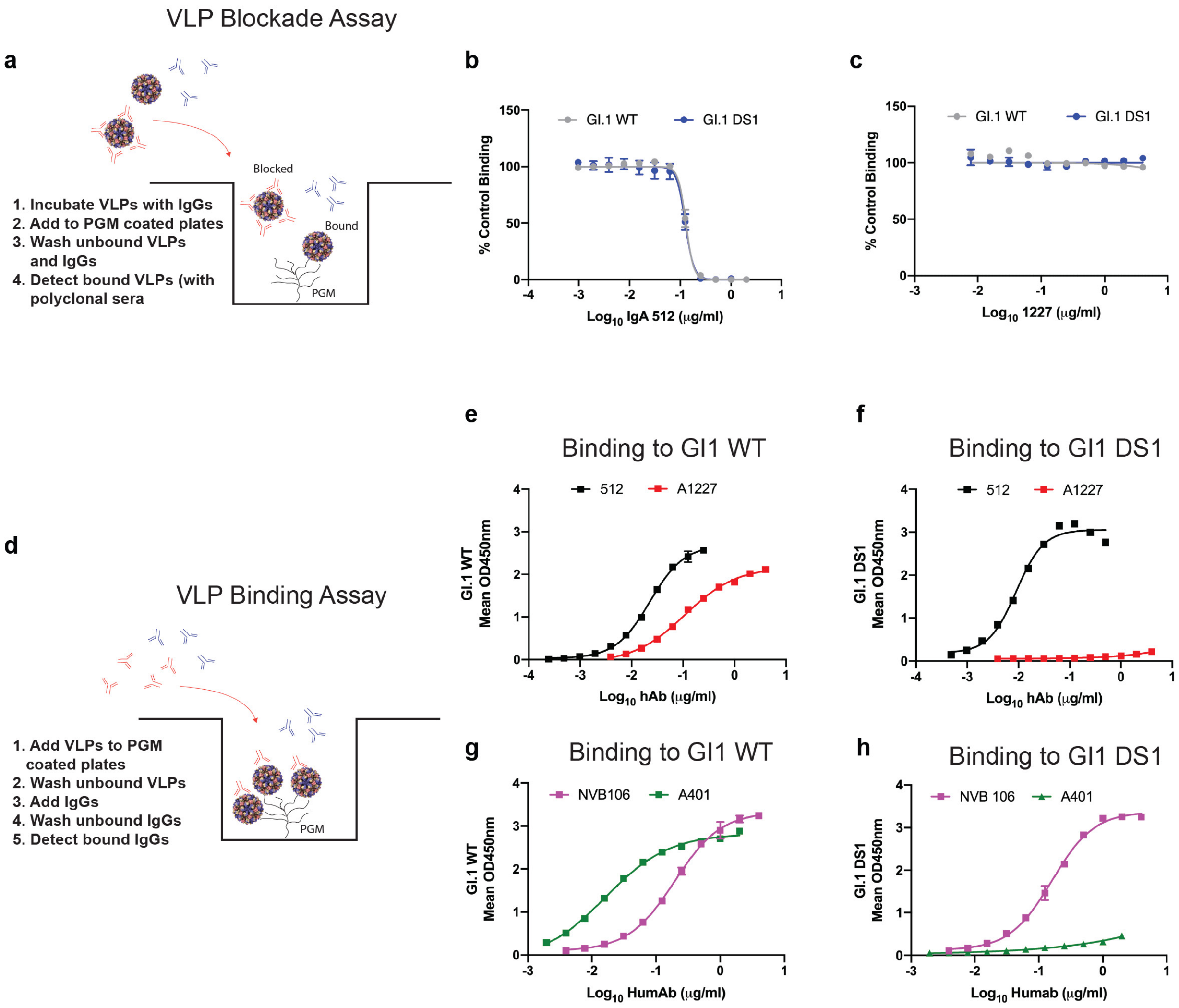

Supplementary Figure 5. Stabilization of GI.1 VLPs preserves neutralizing epitopes but prevents binding of non-blockade antibodies. (a) Schematic of the HBGA (Hist blood group antigen) blockade assay used. (b) Blockade assay using 512 IgG and (c) 1227 IgG. As expected, 1227 is not able to block the binding of GI.1 wild-type or GI.1-DS1 to pig gastric mucin coated plates. 512 IgG was able to block both GI.1 wild-type and DS1 with similar potency. (d) Schematic of the VLP binding assay used. Binding of VLPs to monoclonal antibodies 512 IgA and 1227 IgG was tested by capturing GI.1 wild-type (e) or GI.1-DS1 VLPs (f) onto pig gastric mucin coated plates and incubating the VLPs with 512 or A1227. Notice that A1227 only binds to GI.1 wild-type VLPs, but no detectable binding is measured to disulfide-stabilized particles. Conversely, 512 IgA can bind to GI.1 WT and GI.1-DS1 with similar affinities. (g) GI.1 WT captured on PGM coated plates were incubated with the neutralizing antibody NVB106 and cross-reactive non-neutralizing A401. Both antibodies can bind to the VLPs. (h) The same experiment in (g) was repeated using GI.1 DS1 VLPs. Very low binding of A401 was observed, while NVB106 did not show any difference in binding.

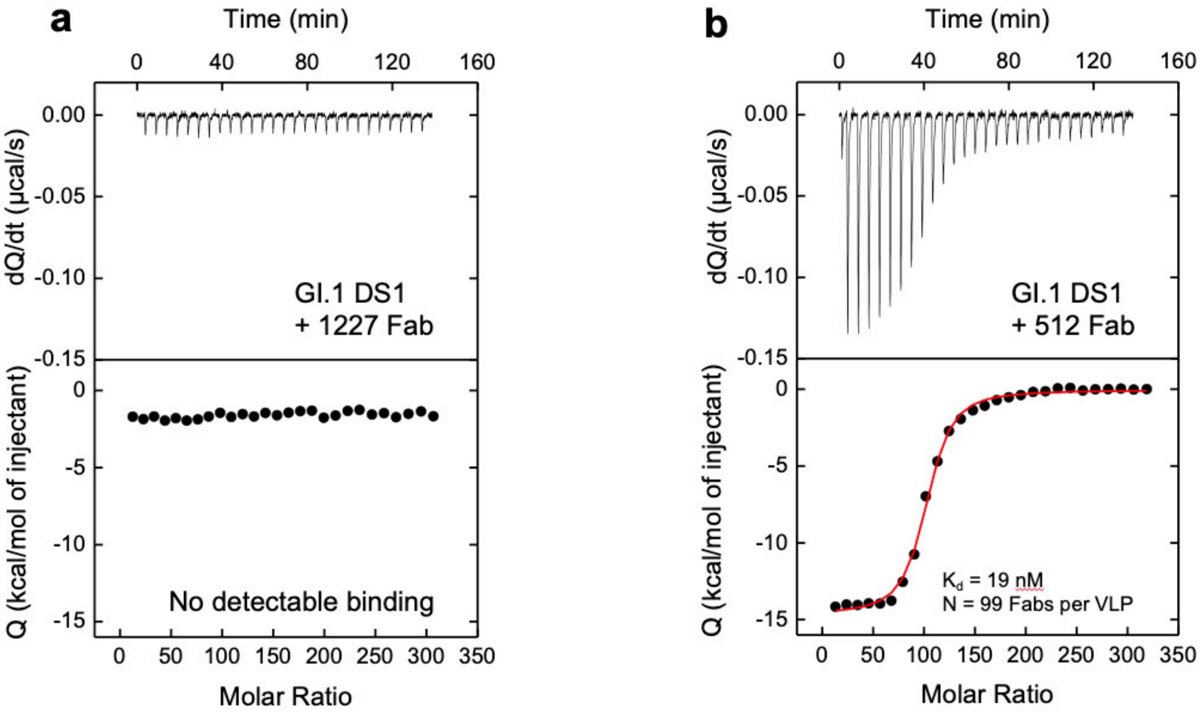

Supplementary Figure 6. Isothermal titration calorimetry shows no detectable binding of A1227 Fab to stabilized particles but high affinity for 512 Fab. Purified GI.1 DS1 VLPs were titrated with A1227 Fab (a) or 512 Fab (b). Notice that no detectable binding was observed when A1227 was titrated into GI.1-DS1.

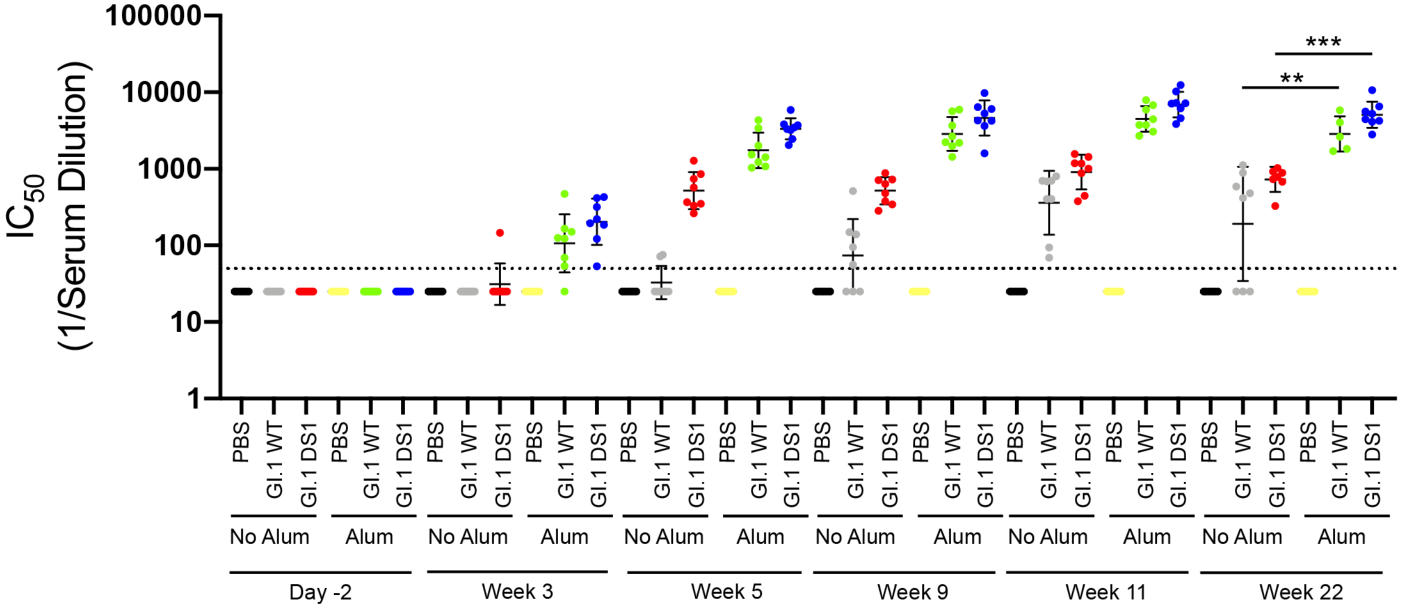

Supplementary Figure 7. HBGA blockade titers for all groups at all time points. Comparison of blockade antibody titers between each group in the presence and absence of alum. Groups are displayed according to blood draw week. Limit of detection (titer = 80) is indicated with a horizontal dotted line. Geometric mean titers ± geometric SD are shown in scatter dot plot. P values were determined by two-tailed Mann-Whitney tests. ** indicates $P \leq 0.01$, *** indicates $P \leq 0.001$.

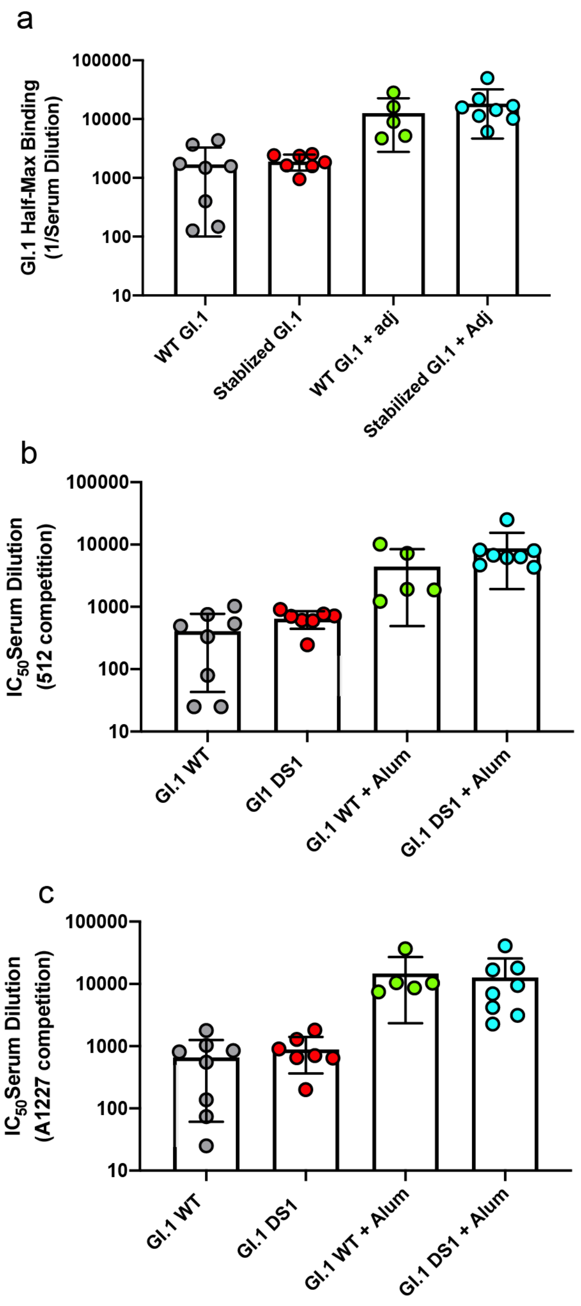

Supplementary Figure 8. GI.1 reactive serum tiers and serum dilution that blocks 512 or A1227 binding. (a) Half-maximum binding titers for all groups. (b) Serum dilution that competes with 512 binding to GI.1 VLPs. (c) Serum dilution that competes with A1227 binding to GI.1 VLPs. In both cases, the 512 or A1227 IgGs were added at a concentration required to achieve 50% maximal binding [EC50]. All plots show box plots with mean ± SD.

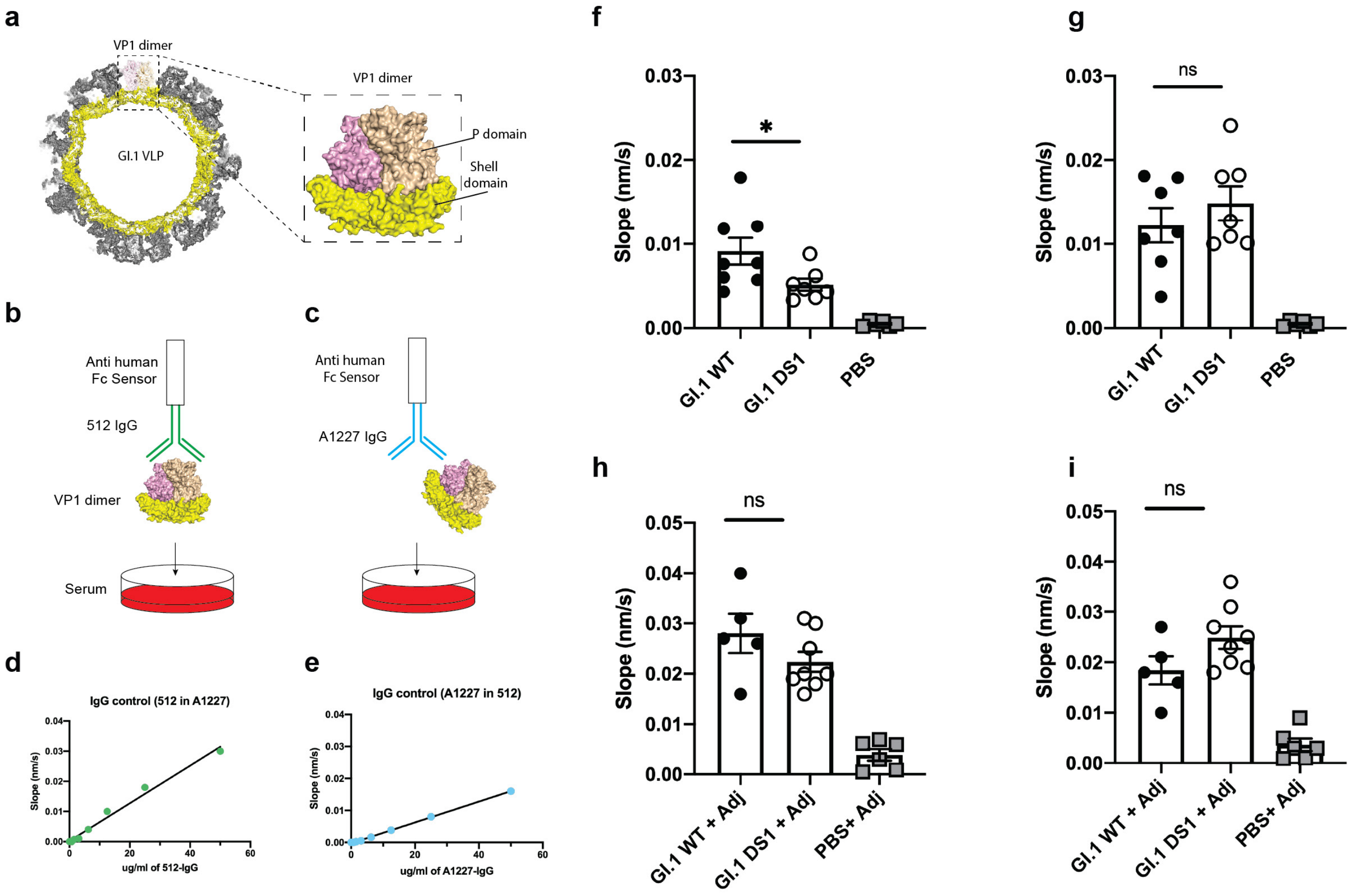

Supplementary Figure 9. Residual binding titers after capturing VP1 dimer with 512 or A1227 IgG. (a) Surface representation of a slice of GI.1 VLP with VP1 dimer shown in the dashed box. For this experiment, dissociated VP1 dimers were purified by size-exclusion chromatography. (b) Schematic of biolayer interferometry assay used to measure residual serum-antibody binding to VP1 dimers captured with 512-IgG or (b) A1227-IgG. Linearity of initial slope measurements was assessed by measuring binding of (d) A1227-IgG to VP1 dimer captured with 512-IgG or (e) 512-IgG to VP1 dimer captured with A1227-IgG. (f) Residual binding of sera at week 22 (50-fold dilution) from mice immunized with GI.1 WT or GI.1 DS1 to VP1 dimer captured with 512-IgG. (g) Same experiment as in (f), but using VP1 dimer captured with A1227-IgG. (h,i) Same experiment as in (f,g), but with sera from adjuvanted groups. PBS controls correspond to sera from mice immunized with PBS or PBS + adjuvant. In all cases, the initial slope of each curve is plotted. Average slopes ± SEM are shown in each box plot. P values were determined by two-tailed Mann-Whitney tests. * indicates $P \leq 0.05$. ns = non significant.

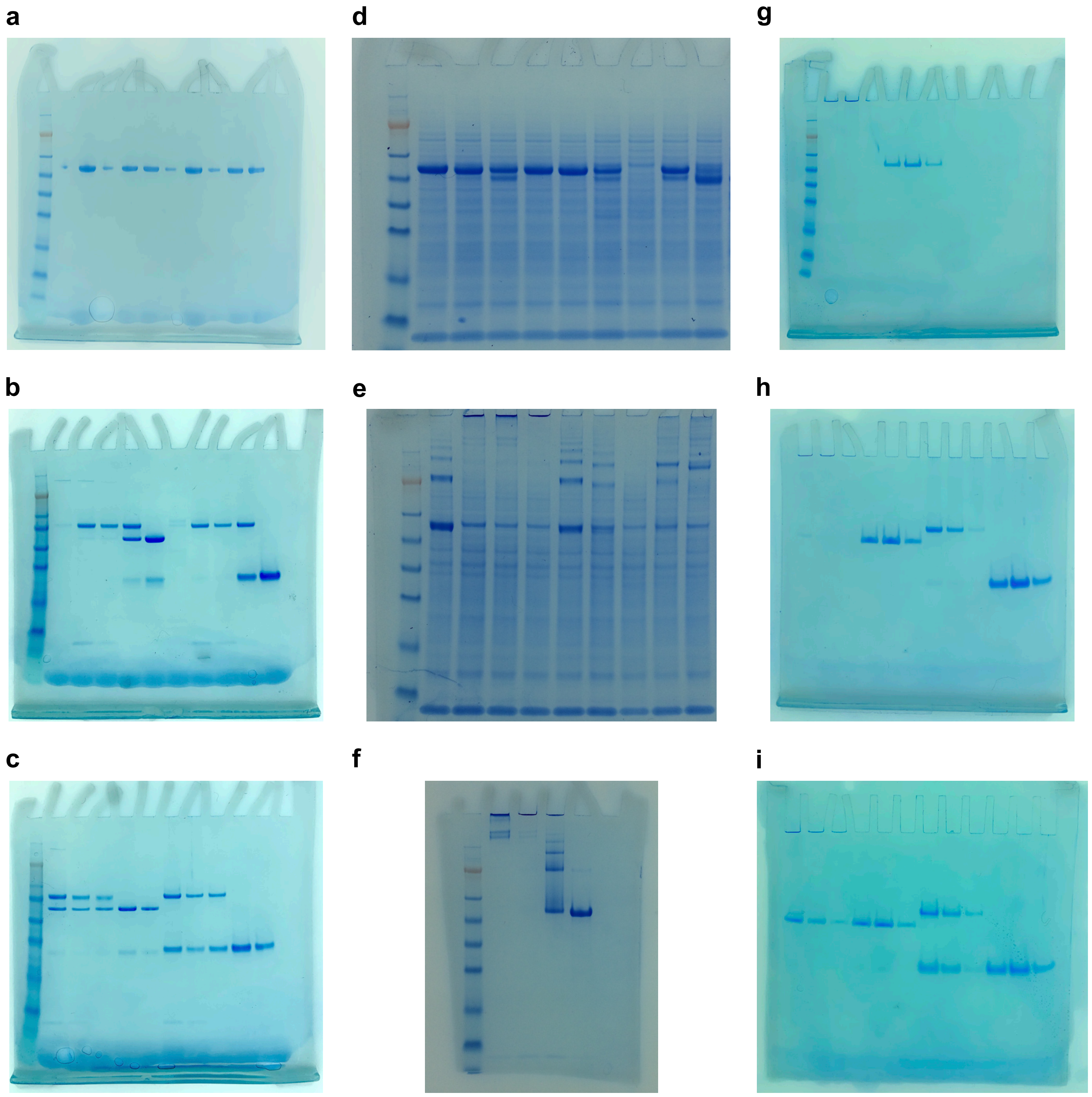

Supplementary Figure 10. Uncropped pictures of the gels presented in Figures 1, 2, and 4. (a-c) Uncropped gels presented in figure 1 g-i. (d-f) Uncropped gels presented in figures 2b and 2e. (g-i) Uncropped gels presented in figure 4 g-i). All gels were run using NuPAGE 4-12% Bis-Tris in MES buffer at room temperature and 200V. SeeBlue™ Plus2 Pre-stained Protein Standards were included in the first lane of each gel.
